# Trained Immunity: An Underlying Driver of Inflammatory Atherosclerosis

**DOI:** 10.3389/fimmu.2020.00284

**Published:** 2020-02-21

**Authors:** Chao Zhong, Xiaofeng Yang, Yulin Feng, Jun Yu

**Affiliations:** ^1^Key Laboratory for Pharmacology and Translational Research of Traditional Chinese Medicine of Nanchang, Center for Translational Medicine, School of Chinese Medicine, Jiangxi University of Traditional Chinese Medicine, Nanchang, China; ^2^Center for Metabolic Disease Research, Department of Physiology, Lewis Katz School of Medicine, Temple University, Philadelphia, PA, United States; ^3^National Pharmaceutical Engineering Center, Jiangxi University of Traditional Chinese Medicine, Nanchang, China

**Keywords:** atherosclerosis, cardiovascular diseases, non-resolving low-grade inflammation, innate immune system, trained immunity

## Abstract

Atherosclerosis, a chronic inflammatory disease of the arterial wall, is among the leading causes of morbidity and mortality worldwide. The persistence of low-grade vascular inflammation has been considered to fuel the development of atherosclerosis. However, fundamental mechanistic understanding of the establishment of non-resolving low-grade inflammation is lacking, and a large number of atherosclerosis-related cardiovascular complications cannot be prevented by current therapeutic regimens. Trained immunity is an emerging new concept describing a prolonged hyperactivation of the innate immune system after exposure to certain stimuli, leading to an augmented immune response to a secondary stimulus. While it exerts beneficial effects for host defense against invading pathogens, uncontrolled persistent innate immune activation causes chronic inflammatory diseases. In light of the above, the long-term over-activation of the innate immune system conferred by trained immunity has been recently hypothesized to serve as a link between non-resolving vascular inflammation and atherosclerosis. Here, we provide an overview of current knowledge on trained immunity triggered by various exogenous and endogenous inducers, with particular emphasis on its pro-atherogenic effects and the underlying intracellular mechanisms that act at both the cellular level and systems level. We also discuss how trained immunity could be mechanistically linked to atherosclerosis from both preclinical and clinical perspectives. This review details the mechanisms underlying the induction of trained immunity by different stimuli, and highlights that the intracellular training programs can be different, though partly overlapping, depending on the stimulus and the biological system. Thus, clinical investigation of risk factor specific innate immune memory is necessary for future use of trained immunity-based therapy in atherosclerosis.

## Introduction

Atherosclerosis, the major contributor for cardiovascular diseases, has been one of the leading causes of morbidity and mortality globally (1, 2). In essence, atherosclerosis is a chronic inflammatory disorder of the arterial wall where non-resolving low-grade inflammation plays a critical role in disease initiation, progression, and final thrombotic complications. Previously, it has been reported that monocytes/macrophages act as a central mediator of inflammatory atherosclerosis. After accumulation of lipoproteins in the vessel wall, the circulating monocytes migrate through activated vascular endothelial cells and then differentiate into macrophages that subsequently uptake modified lipoproteins such as oxidized low-density lipoprotein (oxLDL). With the cholesterol-laden foam cell formation, which is a hallmark of atherosclerosis, a series of complex inflammatory cascades are triggered, thereby promoting atherosclerotic lesion development and ultimately leading to plaque rupture and its related cardiovascular events (3–5). Recent intervention trials that reduced inflammation showed significantly improved cardiovascular outcomes among patients at cardiovascular risk (6, 7), further demonstrating the causal role for inflammation in atherosclerotic cardiovascular disease (ASCVD). Therefore, therapeutically targeting inflammation is considered as a promising strategy to treat atherosclerosis (4). To date, a wide array of factors has been associated with the pathogenesis of atherosclerosis. These factors include not only traditional risk factors such as dyslipidemia, obesity, diabetes, hypertension, aging, and smoking, but also non-classical risk factors such as infections and chronic inflammatory diseases (8). However, how these risk factors lead to the establishment of the non-resolving vascular inflammation during atherogenesis remains unclear. Thus, further unravel the associated mechanisms is of fundamental importance for therapeutic intervention of atherosclerosis.

The innate immune system, which includes monocytes and macrophages, plays a fundamental role in the preservation of immune homeostasis by eliminating infectious agents and promoting tissue damage repair. However, under pathological conditions as mentioned above, it is also responsible for chronic inflammatory diseases such as atherosclerosis. In recent years, a growing body of evidence has demonstrated that the innate immune system can build immunological memory of the past foreign encounters (9–11), challenging the traditional dogma that immunological memory exists only in adaptive immunity. This innate immunological memory, which is also called trained immunity, leads to an augmented immune response to a secondary stimulus through metabolic and epigenetic rewiring of innate immune cells. Importantly, this enhanced innate immune response is non-specific and the trained immunity function is long-lasting, that is the trained immune cells adopt a prolonged activated phenotype that can respond strongly to a wide range of subsequent similar or unrelated stimuli. Therefore, the trained immunity is likely to be an evolutionarily conserved immune mechanism for host defense. Nonetheless, it is of crucial importance to note that trained immunity is a double-edged sword. Uncontrolled hyperactivated immune response conferred by trained immunity could be detrimental, leading to a variety of chronic inflammatory disorders (12). In terms of atherosclerosis which is characterized by non-resolving vascular inflammation, the persistent over-activation of the innate immune system in trained immunity has been recently hypothesized as a mechanism linking non-resolving inflammation and atherosclerosis, indicating the potential causal role of trained immunity in atherogenesis (8, 13–17). A more comprehensive understanding of the mechanisms underlying trained immunity in relation to atherosclerosis would provide additional targets for the prevention and treatment of ASCVD.

## Pro-Atherogenic Effect of Trained Immunity

Atherosclerosis is characterized by non-resolving arterial inflammation in which monocytes/macrophages play a central role, and any factors that promote this inflammation can significantly enhance the development of atherosclerosis. Meanwhile, the persistent activated phenotype of innate immune cells resulting in an exacerbated pro-inflammatory response is a hallmark of trained immunity. Using experimental models of trained immunity, it has been shown that cells related to atherosclerosis can be trained to develop a long-term pro-inflammatory and pro-atherogenic phenotype. In this regard, it is reasoned that trained immunity can act as an important driver of inflammatory atherosclerosis.

In the past several years, considerable studies have validated trained immunity in monocytes and macrophages. By pre-exposure to various primary stimuli such as Bacille Calmette-Guérin (BCG) vaccination, microorganisms, and microbial products, the monocytes/macrophages have been shown to build a long-lasting memory of this encounter leading to an enhanced pro-inflammatory response upon a subsequent challenge, and the resultant protective effect against reinfection is independent of adaptive immune system (18–22). While these studies highlighted heightened host defense against invading pathogens, they provide a key insight into the potential role of trained immunity in infection-related ASCVD given the amplified pro-inflammatory response conferred by trained immunity. As is shown by accumulating reports, bacterial and viral infections are closely related to an increased risk of ASCVD (23–25). Although the mechanism of infection-related ASCVD remains poorly understood, trained immunity has recently been proposed to link infection and the increased incidence of ASCVD (14). It has been well-established that epigenetic modifications at the level of histone methylation and acetylation act as important regulators of trained immunity (10, 11). In support of the potential role of infection-induced trained immunity in atherogenesis, a previous analysis of top 500 genes with enriched histone mark H3K4me3 in microbial constituent (β-glucan) trained macrophages identified a series of genes that are related to the development of atherosclerosis, ranging from pro-atherogenic genes encoding cytokines and chemokines to genes responsible for foam cell formation and plaque instability (26). Moreover, in a mouse model of atherosclerosis (ApoE^−/−^ mice), short-term treatment of lipopolysaccharide (LPS) in a super-low-dose that mimics chronic infection elicits the polarization of monocytes into a sustained pro-inflammatory state with upregulated Ly6C, CCR5, MCP-1, and decreased SR-B1 expression level, and the result of which is the aggravation of atherosclerosis (27). In addition to microbial stimuli, recent findings demonstrated that several non-microbial endogenous products, which have been associated with ASCVD, can also induce trained immunity in monocytes/macrophages (26, 28–30). Notably, training of monocytes by these compounds including oxLDL, lipoprotein(a), aldosterone, and the oligomeric S100A4 induces a pro-inflammatory and pro-atherogenic phenotype. Firstly, the primed monocytes show an enhanced ability of vascular endothelial adhesion and transmigration (28) as well as an increased production of pro-inflammatory cytokines upon re-stimulation by Toll-like receptor (TLR) agonists (26, 28–30). Besides, the cholesterol-laden foam cell formation and the expression of matrix metalloproteinases, which have been implicated in the progression of atherosclerosis, are both significantly accelerated in oxLDL-trained macrophages (26). Of note, a similar trained pro-inflammatory phenotype is also observed in circulating monocytes from patients with symptomatic atherosclerosis (31). The findings from these studies suggest that endogenous pro-atherogenic compounds could contribute to atherosclerosis, at least in part, through trained-immunity-related functional reprogramming of monocytes/macrophages. Collectively, both microbial and sterile endogenous inflammatory triggers can induce trained immunity in monocytes/macrophages that is potentially linked to atherosclerosis. Importantly, as the trained immune phenotype in these studies can persist for prolonged time periods, they also provide an important mechanism responsible for the persistence of vascular inflammation which is the inherent nature of atherosclerosis.

The long-term hyperactivation of innate immune system is the characteristic of trained immunity which can last up to months (18). In contrast, monocytes have a shorter lifespan than the duration of trained immunity. Therefore, it is not sufficient to explain the prolonged trained immunity function at the level of monocytes/macrophages. Currently, emerging evidence has extended the concept of trained immunity and indicated that the bone marrow progenitor cells are integral to the establishment of trained immunity (32–34). Since the bone marrow progenitors are capable of generating lineages of blood cells including monocytes/macrophages, persistent reprogramming of these bone marrow cells to imprint differentiated innate immune cells for a hyper-responsive state is likely to be a general mechanism for the long-term effect of trained immunity. Indeed, in recent studies, administration of BCG vaccination or β-glucan *in vivo* promotes expansion of hematopoietic stem and progenitor cells (HSPCs) and enhances myeloid differentiation through sustained transcriptional and metabolic modulation (32, 33). Importantly, these primed HSPCs give rise to monocytes and macrophages that are programmed to more potently respond to future inflammatory triggers, ultimately providing long-lived enhanced protection against secondary challenges (32). This indicates that training effect can be initiated at the level of bone marrow progenitors, which is then transferred to differentiated myeloid cells and enables these cells to mount enhanced innate immune responses. To this end, it is tempting to expect that similar mechanism could also be applied to atherogenesis and its associated chronic vascular inflammation. A recent study using an atherosclerosis mouse model (LDLR^−/−^ mice) showed that the sterile inflammatory Western diet (WD), which has been known as a risk factor of ASCVD, can similarly trigger hematopoietic precursor cell expansion and myelopoiesis associated with long-term transcriptional and epigenetic reprogramming of myeloid progenitor cells (34). The resulting monocytes from WD-fed LDLR^−/−^ mice display an activation state with augmented immune responses upon TLR stimulation, and this still persists for 4 weeks after switching to chow diet indicating the induction of trained immunity (34). Therefore, although the direct WD-induced training effect on atherosclerosis remains to be investigated, it is logical to speculate that this increased abundance of primed inflammatory monocytes, which were derived from reprogrammed bone marrow progenitors, are likely to promote atherosclerosis development. Indeed, transplantation of the bone marrow from WD-fed LDLR^−/−^ mice into chow-fed recipients has been previously reported to cause a significant increase in atherosclerotic lesion size without affecting serum cholesterol level (35). Another compelling evidence is the hypercholesterolemia-induced aggravation of atherosclerosis, which also involves bone marrow HSPC modulation and probably trained immunity (36). Specifically, hypercholesterolemia induces proliferation and myeloid skewing of HSPCs, and this priming effect of HSPCs persists even after shift to normocholesterolemic bone marrow microenvironment (36). Additionally, monocytes/macrophages that arise from these primed HSPCs show a pro-inflammatory and pro-atherogenic phenotype that is characterized by an increased production of TNF-α, IL-6, MCP-1, and accelerated migration into the artery, eventually resulting in aggravated atherosclerosis (36).

In addition to monocytes/macrophages which are the driving force of atherosclerosis, vascular non-immune cells are also involved in this chronic inflammatory disease. It has been known that both vascular endothelial cells and smooth muscle cells transform into an activation state in the context of vascular inflammation, thus contributing to atherogenesis (4). In fact, these vascular cells retain a high cellular plasticity that is responsive to certain physiological or pathological conditions (37, 38). Interestingly, endothelial cells are now acknowledged as conditional innate immune cells, when activated under certain circumstances, they show characteristics similar to macrophages on many aspects including cytokine secretion, recognition of pathogen-associated molecular patterns (PAMPs), and damage-associated molecular patterns (DAMPs), phagocytic function, and antigen presentation (37). Besides, smooth muscle cells can also recognize DAMPs such as oxLDL to transit to a pro-inflammatory phenotype through TLRs (39, 40), and even in the setting of cholesterol accumulation, they can directly transdifferentiate into macrophage-like cells that enhance atherogenesis (41). Therefore, endothelial cells and smooth muscle cells play a dynamic and meaningful role in immune function. Currently, accumulating data suggest that these vascular non-immune cells can also be trained to develop a prolonged memory. It has been previously reported that short-term high glucose induces transition of aortic endothelial cells to a long-lived pro-inflammatory status with elevated expression of NF-κB and its downstream pro-atherogenic genes MCP-1 and VCAM-1 both *in vitro* and *in vivo* (42), extending the concept of memory training to non-immune endothelial cells. More recently, it was shown that the pro-atherogenic lipid molecule lysophosphatidylcholine (LPC) induces reprogramming of aortic endothelial cells for sustained pro-inflammatory responses (43). Further integrated transcriptomic and epigenetic analysis revealed that LPC induces metabolic and epigenetic changes in activated endothelial cells, including pathways known to be pivotal for the induction of trained immunity (44). These studies provide a novel working model by which endogenous pro-atherogenic compound induces training and long-term activation of endothelial cells during atherogenesis. They also suggest that in this process, memory developed by endothelial cells plays a contributory role in maintaining their prolonged activation state, thereby promoting vascular inflammation during atherosclerosis development. As an important player in atherosclerosis, smooth muscle cells have also been implicated in carrying a memory. For example, vascular smooth muscle cells isolated from a mouse model of diabetes display a potential pro-atherogenic phenotype such as upregulated inflammatory gene expression, accelerated migration ability, and enhanced adhesion to monocytes, which persists *ex vivo* for relatively long periods (45). In addition, brief oxLDL stimulation also induces a persistent pro-inflammatory priming effect in cultured coronary smooth muscle cells (46). Interestingly, the mechanisms involved in this process were found to be somewhat similar to trained immunity induced in myeloid cells (46). Altogether, the establishment of a memory for previous encounters is an important trait of activated endothelial cells and smooth muscle cells in response to pro-atherogenic stimulation. Based on the activation phenotype as described above, it is not surprising that by accelerating the recruitment of immune cells and enhancing the pro-inflammatory responses in the arterial wall, these trained vascular non-immune cells may play a significant role in the vascular inflammation and atherogenesis *in vivo*.

Taken together, trained-immunity-mediated pro-atherogenic effect has now gained growing appreciation. Mature myeloid cells (monocytes/macrophages), bone marrow progenitor cells (hematopoietic and myeloid progenitor cells), and vascular non-immune cells (endothelial cells and smooth muscle cells) have been identified to be integral to trained immunity that potentially drives vascular inflammation. Trained immunity carried by these various cell types are likely to act cooperatively *in vivo* to promote the development and progression of atherosclerosis.

## Mechanisms of Trained Immunity: A Comprehensive Integration of Signaling, Metabolic, and Epigenetic Events

With the establishment of the emerging concept of trained immunity, considerable studies have focused on its potential mechanisms. It is currently believed that trained immunity is driven by a series of comprehensive signaling, metabolic, and epigenetic processes. For an individual myeloid cell, immunological challenges are first sensed by certain receptors, triggering specific trained-immunity-inducing signaling pathways that subsequently mediate long-term metabolic and epigenetic adaptations, eventually leading to a heightened innate immune response upon re-stimulation. On a systems level, stimulated bone marrow progenitors undergo transcriptional, metabolic, and epigenetic remodeling, which facilitates their more robust secondary response, as well as that of their progeny cells, to subsequent challenges. In addition, recent findings also suggested that the induction of cellular memory in non-immune cells may share similar mechanisms with innate immune training (46). While a variety of exogenous and endogenous stimuli can induce trained immunity, our current knowledge about the underlying mechanisms is mainly based on studies investigating the induction of trained immunity by several model training stimuli including β-glucan, BCG vaccination, and oxLDL. Depending on the initial stimulus and the cellular context, the induced intracellular training programs can vary. In this section, we will review the current understanding of the mechanisms regulating trained immunity. As trained immunity has broad implications for atherosclerosis as previously described, these mechanistic knowledges provide important insights into how trained immunity is potentially linked to atherosclerosis and new strategies for treating ASCVD from a perspective of targeting trained immunity.

### β-Glucan-Induced Training Program

β-glucan, a major cell wall component of fungi, is one of the most widely used model stimuli in studying trained immunity. Mechanistically, β-glucan is recognized by the transmembrane C-type lectin receptor dectin-1, which then initiates the complex intracellular training events (19). Building upon this, several studies have been undertaken to characterize the induced biological processes during monocyte training by β-glucan. An initial study identified the cellular metabolic switch from oxidative phosphorylation to glycolysis, which is mediated by AKT-mTOR-HIF1α signaling, as a key metabolic basis for β-glucan-induced trained immunity in monocytes, providing the energy and intermediate metabolites for innate immune cell activation (47). Blockade of either glycolysis or the mTOR signaling using pharmacological or genetic approach inhibits trained immunity both *in vitro* and *in vivo*, indicating the essential role of mTOR signaling-mediated glycolysis in inducing trained immunity (47). In a subsequent study, a combination of transcriptomic and metabolomic analysis further revealed that the glycolysis, glutaminolysis, together with cholesterol synthesis are three major non-redundant metabolic pathways required for β-glucan-induced training of monocytes, illustrated by inhibition of trained immunity induction when any one of these pathways is blocked (48). Notably, crosstalk between these key metabolic pathways is also indicated in orchestrating β-glucan-induced training process. For example, it was recently shown that mevalonate, a key intermediate metabolite of cholesterol synthesis pathway, modulates and induces trained immunity via promoting IGF1-R pathway and its downstream mTOR signaling-mediated glycolysis (49). The activated glycolysis would in turn facilitate mevalonate production via tricarboxylic acid (TCA) cycle, generating a positive feedback loop that strengthens the trained-immunity-inducing effect (49). In addition, pentose phosphate pathway and fatty acid synthesis are also upregulated during training with β-glucan, but neither of them seems to be indispensable for the induction of trained immunity (48).

On the other hand, as a regulatory means of gene expression, epigenetic reprogramming especially the histone modification is also an important mechanism driving trained immunity. It has been shown that β-glucan induces genome-wide epigenetic rewiring of the histone marks H3K4me1, H3K4me3, and H3K27ac in the *in vitro* model of trained immunity in monocytes, which is closely associated with the regulation of trained-immunity-related innate immune and signaling pathways (19, 50). These epigenetic adaptations persist over time after the removal of the initial training stimulus and prime target cells to respond more vigorously to future challenges (e.g., an enhanced expression of pro-inflammatory genes upon re-stimulation). Importantly, pharmacological inhibition of histone methyltransferase represses trained immunity induced by β-glucan (19, 47). As two major cellular events involved in trained immunity, the epigenetic reprogramming is intertwined with the metabolic rewiring, forming epigeno-metabolic circuits. For example, genes involved in glycolysis undergo histone modifications in β-glucan-trained macrophages, indicating the role of epigenetic mechanism in cellular metabolic regulation during innate immune training (47). Concurrently, cellular metabolism also directly influences epigenetic reprogramming in the induction of trained immunity. One clear example is the inhibition of histone demethylase KDM5 and the resulting increase in H3K4me3 due to accumulation of fumarate which is a key metabolite in mediating β-glucan-induced trained immunity in monocytes (48). Moreover, a recent finding revealed the role of long non-coding RNAs (lncRNAs) in generating H3K4me3 in β-glucan-trained macrophages, providing a novel mechanism regulating epigenetic remodeling in trained immunity (51).

Apart from metabolic and epigenetic regulation, the induction of trained immunity is also modulated at the level of signaling mechanism. It has been reported that trained immunity induced by β-glucan in monocytes is critically dependent on cAMP-PKA, IL-1, and IL-32 signaling induction (50, 52). Using pharmacological blockade or functional genomic analysis, it was shown that disruption of cAMP-PKA or IL-1 signaling inhibits training capacity of monocytes while enhancing IL-32 expression accelerates β-glucan-induced trained immunity (50, 52). Interestingly, IL-1 and IL-32 can promote the expression of each other in monocytes during β-glucan training, thus forming a self-reinforcing activating mechanism by which training effect of β-glucan can be enhanced (52). Given the previously described role of IL-1 and IL-32 in metabolic regulation (33, 53), it would be of potential interest to investigate whether they also contribute to metabolic adaptations essential for β-glucan-induced training of monocytes.

In addition to act directly in mature myeloid cells, β-glucan-induced training can be initiated in bone marrow progenitors which results in the expansion of myeloid-biased HSPCs associated with a long-term beneficial response to a secondary systemic inflammatory challenge and protection against chemotherapy-induced myeloablation (33). An integrated transcriptomic, metabolomic, and lipidomic analysis of HSPCs upon β-glucan training demonstrated profound changes in glucose and lipid metabolism such as increased glycolysis and cholesterol synthesis especially the mevalonate pathway (33). The causal role of glycolysis and cholesterol synthesis in β-glucan-induced training in HSPCs is indicated by a reduction of myelopoiesis resulting from pharmacological inhibition of either metabolic pathway (33). Notably, glycolysis and cholesterol synthesis are also both important for β-glucan-induced trained immunity in mature myeloid cells (47, 48). Furthermore, it was found that β-glucan-dependent myelopoiesis requires IL-1 and GM-CSF signaling as well (33). Interestingly, these signaling events are interconnected with the aforementioned cellular metabolic changes, as evidenced by the role of IL-1 signaling in promoting glycolysis and an increased surface expression of CD131 (the common β subunit of the IL-3/GM-CSF receptor) due to accumulation of cholesterol esters in HSPCs during β-glucan-induced myelopoiesis (33).

Of note, in the context of β-glucan-mediated training in monocytes, whole-genome analysis of H3K4me3 demonstrated that many genes involved in the pathogenesis of atherosclerosis are epigenetically primed for an activation status (26). These genes encode not only pro-atherogenic cytokines and chemokines, but also proteins related to foam cell formation and plaque vulnerability (26), further suggesting that the mechanism driving trained immunity can exert pro-atherogenic effects and may contribute to ASCVD. Thus, the well-characterized mechanism of β-glucan-mediated trained immunity can serve as a foundation for our better understanding of how trained immunity potentially drives atherosclerosis.

### BCG-Induced Training Program

The BCG vaccination, which exerts beneficial non-specific effects against infections through a process termed trained immunity, has been established as a model stimulus for trained-immunity-related studies. Mediated by cytoplasmic pattern recognition receptor NOD2 in monocytes (18), BCG-induced training program shows a high similarity to that induced by β-glucan. In terms of metabolic adaptations, BCG-trained macrophages display a metabolic switch with increased glycolysis, glutaminolysis, and pentose phosphate pathway comparable to that of β-glucan-trained macrophages (54). Nonetheless, BCG training does not lead to the classical Warburg effect seen in β-glucan-trained macrophages because there is an upregulation of both glycolysis and oxidative phosphorylation upon BCG-induced training (54). Using the *in vitro* and *in vivo* models of trained immunity, it was shown that mTOR signaling-mediated glycolysis, glutamine metabolism, and cholesterol synthesis known to be required for β-glucan training are also essential for BCG-induced trained immunity (49, 54). With regard to epigenetic rewiring, the epigenetic profile induced by BCG involves remodeling of the histone marks H3K9me3, H3K4me3, and H3K27ac (18, 22, 54), with at least the latter two also occur in β-glucan-induced trained immunity. These epigenetic changes are extensively involved in the regulation of signaling- and inflammatory-related pathways and are indispensable for trained immunity induced by BCG (18, 22). Like in β-glucan-trained macrophages, the metabolic and epigenetic rewiring in BCG-trained macrophages shows a high dependency on each other as well, altering one process can influence the other (54). Moreover, it was shown that IL-1 pathway similarly plays a key role in BCG-induced trained immunity in human monocytes (22). Genetic variations in genes encoding components of IL-1 pathway such as IL-1β, IL-1 receptors, IL-18 receptors, and inflammasome-associated PYCARD/ASC significantly affect trained immunity responses induced by BCG (22). Intriguingly, in mice, the generation of BCG-mediated trained macrophages requires IFN-γ signaling instead of IL-1 signaling (32).

It has been reported that the bone marrow progenitors can be trained by BCG to undergo enhanced expansion and myelopoiesis via long-term transcriptional reprogramming, a phenomenon similarly observed in β-glucan-induced training in the bone marrow (32, 33). Transcriptomic analysis further identified IFN-γ signaling as a required pathway for BCG-dependent HSPC expansion and myelopoiesis, contrasting the critical role of IL-1 and GM-CSF signaling in β-glucan-induced myelopoiesis (32, 33). More recently, IL-32 was revealed as an additional important regulator for BCG-induced training in the bone marrow. Using transcriptomic approach, it was found that IL-32 regulates genes involved in cell metabolism, inflammatory immune response, transcriptional regulation, and signaling transduction in bone marrow progenitors, which are important for trained immunity induction (52). Notably, BCG-induced cell reprogramming in HSPCs has been demonstrated to be ultimately transmitted to their progeny cells, leading to epigenetically primed macrophages and a subsequent more protective effect against infection (32).

A previous study showed that BCG vaccination has a potential role in promoting atherosclerosis (55). The accelerated atherosclerosis observed in BCG-vaccinated rabbits fed a cholesterol diet, in which plasma cholesterol level keeps unchanged compared with saline injected control group, is associated with increased circulating leucocyte (including monocyte) activation and enhanced aortic monocyte recruitment (55), suggesting that BCG-induced trained immunity may be involved in the atherogenesis process. In line with this, a recent genome-wide H3K27ac chromatin immunoprecipitation sequencing (ChIP-seq) analysis showed that the gene encoding receptor of oxLDL which is a marker of atherosclerosis, as well as genes directly related to inflammation, are enriched with H3K27ac in monocytes upon BCG vaccination (22), indicating that these pro-atherogenic genes are primed for an activation state in BCG-trained monocytes which could in turn contribute to atherosclerosis. However, it is noteworthy that several additional studies showed opposite results, pointing toward a beneficial effect of BCG vaccination on ASCVD (56–58). For example, in a human epidemiological cohort study, children vaccinated with BCG only showed a hazard ratio of 0.36 to develop cardiovascular diseases (58). Accordingly, it has been previously hypothesized that this could be due to the fact that BCG vaccination protects against other infections that themselves would aggravate ASCVD (14).

### oxLDL-Induced Training Program

It has been well-documented that oxLDL acts as a crucial mediator of atherosclerosis by triggering inflammatory cascades important for atherogenesis (59–61). Following the discovery of β-glucan and BCG as microbial training stimuli, the sterile endogenous compound oxLDL was later identified to be able to induce trained immunity as well. Monocytes briefly exposed to a low concentration of oxLDL *in vitro* are programmed toward a pro-atherogenic state with augmented foam cell formation capacity, increased production of matrix metalloproteinases as well as elevated expression of pro-atherogenic cytokines and chemokines in response to TLR re-stimulation (26), indicating a potentially detrimental effect in atherosclerosis *in vivo*. Unlike CD36-mediated innate immune cell activation upon oxLDL stimulation, oxLDL induces training of monocytes in a manner that specifically through TLR pathway which is CD36-independent (26). The oxLDL-trained monocytes and macrophages display some mechanistic features known to be important for β-glucan- and BCG-induced trained immunity. Firstly, a metabolic switch to glycolysis that depends on mTOR signaling similarly occurs in oxLDL-trained macrophages (62, 63). Pharmacological inhibition of mTOR activation blocks not only downstream HIF1α expression and glycolysis but also oxLDL-trained macrophage phenotype (63), indicating the critical role of mTOR signaling in oxLDL-induced training process. However, the potential role of glycolysis in oxLDL-induced trained immunity remains to be determined. Secondly, oxLDL training induces an increase in scavenger receptors CD36, SR-A and a reduction in cholesterol efflux transporters ABCA1 and ABCG1 which is associated with enhanced foam cell formation, suggesting the modulation of cholesterol metabolism in oxLDL-trained cells (26). Illustrating the importance of the cholesterol metabolic pathway, inhibition of cholesterol synthesis by fluvastatin results in the blockade of oxLDL-mediated trained immunity responses (49). Thirdly, monocytes primed with oxLDL undergo epigenetic reprogramming, as evidenced by an enriched histone mark H3K4me3 on genes encoding various pro-atherogenic cytokines, chemokines, and transporters (26). Using the non-specific histone methyltransferase inhibitor, the oxLDL-induced trained immunity is completely abrogated (26). In addition, functional genomic studies showed that the IL-1 pathway, in accordance with its pivotal role in β-glucan- and BCG-induced trained immunity, governs oxLDL-induced training of monocytes as well (34). Nevertheless, oxLDL-induced training program also shows difference compared with that induced by β-glucan. As mentioned above, the mTOR-HIF1α-axis is a shared pathway for trained immunity induced by oxLDL and β-glucan. Recently, it was further reported that the mTOR signaling in monocytes primed with oxLDL promotes reactive oxygen species (ROS) formation which is critical for oxLDL-induced trained immunity (63). By contrast, training of monocytes with β-glucan leads to a decreased production of ROS albeit an activation of mTOR signaling (62). In contrast to the well-characterized β-glucan- and BCG-induced training programs, the intracellular training process mediated by oxLDL remains to be defined. Further studies using transcriptomic, metabolomic, and epigenomic approaches are warranted to comprehensively reveal this process.

oxLDL has been associated with the cholesterol rich WD. Previous studies have shown that WD feeding can induce functional reprogramming associated with epigenetic alterations in the bone marrow, and ultimately generate an abundance of inflammatory monocytes/macrophages that increases the susceptibility to atherosclerosis (35, 36). However, given that the duration of these modifications in bone marrow cells has not been investigated in these studies, whether trained immunity is involved in WD-induced priming of bone marrow progenitors that aggravates atherosclerosis remains unclear. Nonetheless, a more recent study provided evidence that WD can induce trained immunity with a long-lasting priming of the innate immune system even after dietary change, evoking a chronic low-grade inflammatory state (34). In this study, it was shown that WD induces training effect in the innate immune system in a manner comparable to those induced by β-glucan and BCG, causing the expansion and modulation of bone marrow progenitor cells and subsequent generation of activated and potentially pathological innate immune cells (34). Also, the myeloid cell-mediated innate immune responses remain exacerbated upon dietary change (34). These effects are associated with a long-lasting reprogramming of transcriptome and chromatin accessibility landscape related to immune cell development and signaling in myeloid progenitors (34). In addition, WD-induced training of the innate immune system was shown to be NLRP3 inflammasome/IL-1 pathway dependent (34), a classical pathway that is also required for β-glucan-induced innate immune training. Deficiency of *NLRP3* prevents induction of hematopoiesis, reprogramming of bone marrow myeloid progenitors and systemic inflammation in response to WD (34). As we all know, the sterile inflammatory WD has been linked to an increased incidence of ASCVD (64), it is thus conceivable that besides hypercholesterolemia resulting from WD, WD-induced trained immunity is also involved in the development of atherosclerosis. Intriguingly, the histone demethylase *Tet2*, which has been identified as a risk of atherosclerosis associated with dysfunctional myelopoiesis and activated NLRP3 inflammasome/IL-1 pathway (65), was found to be epigenetically primed in myeloid progenitors of WD-fed LDLR^−/−^ mice (34), providing additional evidence that WD-induced trained immunity plays a causal role in atherogenesis. While WD-induced innate immune reprogramming has been extensively characterized using an atheroprone mouse model, it remains to be addressed whether these training effect induced by WD can also occur in wild-type mice.

### LPS-Induced Training Program

Whereas, a high level of bacteria endotoxin LPS has been established to result in innate immune tolerance with an attenuated immune response to a secondary challenge, a subclinical super-low-dose LPS are found to induce a prolonged innate immune activation via a process relevant to trained immunity (27, 66, 67), although they are both mediated by cell surface TLR4. Previous *in vitro* studies have demonstrated the inhibition of homeostatic negative regulators required for immune tolerance such as PI3K and IRAK-M, as well as induction of molecular networks involving IRAK-1 and Tollip, as the underlying mechanism for LPS-induced priming of macrophage activation, leading to a prolonged and mild pro-inflammatory status (66, 67). From a clinical perspective, subclinical endotoxemia resulting from chronic infection or metabolic disorders represents a strong risk factor for the occurrence of chronic inflammatory diseases such as atherosclerosis (68, 69). It is tempting to speculate that trained immunity induced by super-low level of endotoxin contributes to the non-resolving vascular inflammation and atherosclerosis. Indeed, a recent study using ApoE^−/−^ mouse model of atherosclerosis found that short pre-conditioning of super-low-dose LPS induces low-grade inflammation and aggravates atherosclerosis development through priming monocytes into a non-resolving pro-inflammatory phenotype, indicating that super-low-dose LPS induces innate immune memory *in vivo* that exerts pro-atherogenic effect (27). Adoptive transfer of these LPS-primed monocytes to non-LPS-treated mice significantly exacerbates atherosclerosis, further demonstrating the causal role of LPS-induced trained immunity in atherogenesis (27). Further mechanistic studies identified a negative feedback circuit comprised of an interplay between the activated JNK-miR-24 and the reduced Smad4-IRAK-M to be responsible for the long-term activation of pro-inflammatory and pro-atherogenic monocytes by subclinical-dose LPS (27), highlighting the potential of manipulating this negative feedback circuit to suppress the persistence of low-grade inflammation and its pathological relevance in atherosclerosis development. Moreover, in the context of low-dose LPS, the stimulated monocytes show a metabolic rewiring with an increase in both glycolysis and oxidative phosphorylation (70), which is comparable to monocytes primed with BCG, but not to β-glucan or higher dose of LPS that exhibits the Warburg effect. In the meantime, super-low-dose LPS also induces changes in cholesterol metabolism. By reducing the expression of cholesterol transporters SR-B1, ABCA1, and ABCG1, cellular cholesterol efflux from macrophages primed with super-low-dose LPS is suppressed which is associated with elevated foam cell formation *in vitro* and aggravated atherosclerosis *in vivo* (27, 71). However, the role of glucose and cholesterol metabolism in LPS-induced trained immunity remains to be established. Additionally, it has been reported that higher dose of LPS can induce bone marrow HSPC expansion and their myeloid skewing termed emergency myelopoiesis, but more importantly, it is also associated with HSPC functionality impairment (72). By contrast, trained immunity at the level of bone marrow mediated by β-glucan acts favorably on HSPCs and myelopoiesis (33). Thus, it would be of interest to investigate whether subclinical super-low-dose LPS triggering trained immunity can also induce prolonged adaptations in progenitor cells of the innate immune system in the bone marrow without causing their functional exhaustion, thereby generating more abundant non-resolving pro-inflammatory monocytes that promote atherosclerosis.

### Aldosterone-Induced Training Program

Aldosterone is a hormone regulating blood pressure and electrolyte homeostasis. It has been reported that aldosterone is a risk factor of ASCVD independent of its role in causing hypertension: supranormal levels of aldosterone accompany the pro-atherogenic effects while inhibition of the aldosterone pathway blunts the detrimental effects on cardiovascular health (73). More recently, it was shown that brief exposure to high levels of aldosterone in cultured human monocytes induces a prolonged pro-inflammatory state characterized by augmented pro-inflammatory cytokine responses upon re-stimulation, indicating the induction of trained immunity by aldosterone (29). Therefore, aldosterone-induced trained immunity provides a novel potential mechanism linking primary hyperaldosteronism and inflammatory atherosclerosis. Of particular note, via the mineralocorticoid receptor, the aldosterone-induced training program is to a large extent different from that induced by model training ligands such as β-glucan and BCG. Glycolysis has been well-established as the metabolic basis of β-glucan- and BCG-induced trained immunity (47, 48, 54), but strikingly, aldosterone training does not induce change in glycolysis and oxidative phosphorylation (29). Instead, it leads to the upregulation of fatty acid synthesis as well as lipid and glycan metabolism upon re-stimulation (29). Importantly, blockade of fatty acid synthesis abolishes aldosterone-induced trained immunity responses, indicating the fundamental role of fatty acid synthesis in aldosterone-mediated trained immunity (29). Interestingly, fatty acid synthesis is also elevated upon β-glucan training, but it is not necessary for the induction of trained immunity by β-glucan (48). Moreover, aldosterone has been known to enable cholesterol accumulation in innate immune cells that could drive the progression of atherosclerosis (73). Given the importance of cholesterol metabolism in the innate immune training, it would be of potential interest to explore whether it is also modulated during aldosterone-induced training and its role in the induction of aldosterone-mediated trained immunity. Collectively, these findings suggest that aldosterone adopts a largely different immunometabolic route to induce trained immunity as compared with prototypical trained-immunity-inducing agonist β-glucan or BCG. In addition, as an important determinant of trained immunity, epigenetic modifications also occur in aldosterone-trained macrophages as reflected by an enrichment of histone mark H3K4me3 in genes related to fatty acid metabolism and pro-inflammatory cytokines (29), and the same kind of histone modification is also found in β-glucan-, BCG-, and oxLDL-induced trained immunity. Given that aldosterone is associated with an increased cardiovascular risk in human and it can induce training of monocytes *in vitro*, it is tempting to explore aldosterone-induced trained immunity *in vivo* and its consequences for atherosclerosis susceptibility. Strikingly, a more recent study showed that although aldosterone induces enhanced low-grade arterial wall inflammation in patients with primary aldosteronism (PA) compared to hypertensive controls, the circulating monocytes of PA patients do not show cellular reprogramming associated with *in vivo* induction of trained immunity (e.g., an increased cytokine production capacity and an enhanced expression of genes regulating glycolysis, cholesterol or fatty acid metabolism) (74). Therefore, the enhanced arterial inflammation in PA patients is not mediated by trained immunity of monocytes and is hypothesized to be caused by interaction between various cell types that are related to atherogenesis (74).

## Trained Immunity is Mechanistically Linked to Atherosclerosis

The induction of trained immunity is critically mediated by a highly integrated signaling, metabolic, and epigenetic events, during which innate immune cells remodel their metabolism to accommodate the increased energy demands and provide essential building blocks for the acquisition of a trained phenotype, and concurrently, they also reorganize their chromatin architecture to prime genes from a repressed state to an activated state that underlies trained immunity. At a systems level, trained immunity effect can be initiated by metabolic, epigenetic, and transcriptional modulation of bone marrow progenitor cells, leading to their increased expansion and myeloid differentiation, thereby enhancing the development of activated innate immune cells with a trained phenotype. In this regard, trained immunity represents a mechanism responsible for the induction of a prolonged hyperactivation state of the innate immune system, evoking a stronger pro-inflammatory response to a subsequent re-stimulation. This working model of trained immunity shares common mechanistic features with the pathogenesis of atherosclerosis that also largely depends on activation of the innate immune system. Driven by various pro-atherogenic stimuli, not only macrophages in the atherosclerotic plaque but also monocytes in the circulation and progenitors in the bone marrow can also modulate their cellular metabolism and remodel their chromatin structure to adopt an activation state executing a pro-inflammatory and pro-atherogenic effect. The involved intracellular mechanisms of atherogenesis include activation of signaling such as IL-1 and GM-CSF pathways, modulation of cellular metabolism such as glycolysis, cholesterol metabolism, fatty acid synthesis, and amino acid metabolism, as well as reprogramming of various epigenetic signatures. For a detailed overview of these mechanisms controlling innate immune activation in relation to atherosclerosis, we refer to some excellent recent reviews (4, 75–77). Of note, these pro-atherogenic signaling, metabolic, and epigenetic events are also involved in the induction of trained immunity. Manipulation or intervention of these pathways has been shown to profoundly affect atherosclerosis development and trained immunity induction using *in vitro* or experimental animal models. Indeed, epigenetic remodeling in trained immunity has been shown to induce the priming of genes that are involved in different stages of atherosclerosis from the initial foam cell formation to the eventual atherosclerotic plaque rupture, as well as genes directly associated with inflammation (26). Taken together, it is reasoned that these shared mechanisms may provide a link between trained immunity and the pathogenesis of atherosclerosis.

In agreement with the aforementioned notion, there is accumulating clinical data indicating the potential involvement of trained immunity in patients with atherosclerosis. Using the FDG-positron emission tomography (PET) approach, an increased glucose metabolism can be revealed in human atherosclerotic plaques especially regions of inflammatory cell infiltration and lipid-rich necrotic core (78–80). More recently, it was further shown that high-risk vulnerable plaques have a specific metabolic profile that is distinct from the metabolite signature of low-risk stable plaques (81). One of the major differences in the metabolic profile is that the high-risk plaques exhibit an enhanced glycolysis as compared with the stable ones (81). As glycolysis has been known as the metabolic basis for trained immunity (47, 54), it can be thus hypothesized that trained immunity may contribute to the development and progression of atherosclerosis, leading to an aggravated vascular inflammation in the late stage of atherosclerotic lesions, although it remains to be further explored. Indeed, high-risk vulnerable plaques produce a higher level of pro-inflammatory cytokines and chemokines as compared with low-risk stable plaques (81).

The more compelling evidence of the involvement of trained immunity in atherosclerosis comes from the circulating monocytes. It was shown that monocytes in patients with atherosclerotic coronary artery disease are primed for an pro-inflammatory state which can still persist upon their *ex vivo* differentiation into macrophages, leading to a heightened pro-inflammatory response that could drive systemic and vascular inflammation (82). Mechanistically, an increased glycolytic activity was found to serve as a basis for the induction of this pro-inflammatory state (82). Similar pro-inflammatory phenotype are also observed in another study using monocytes from patients with symptomatic coronary atherosclerosis, which is associated with a metabolic shift toward glycolysis, and with epigenetic rewiring of histone marks on pro-inflammatory genes (31). Remarkably, these features are well-consistent with the concept of trained immunity, suggesting that trained immunity is inherently associated with atherosclerosis. Interestingly, development of such a trained phenotype in monocytes is an exclusive characteristic for symptomatic atherosclerosis, but not for mild and asymptomatic atherosclerosis, supporting the hypothesis that trained immunity may drive the progression of atherosclerosis toward a severe symptomatic disease stage (31). In addition, monocytes from patients with risk factors of ASCVD such as elevated circulating levels of lipoprotein(a) and hypercholesterolemia also display a trained immunity phenotype with an enhanced capacity of cytokine production (28, 83) and an accelerated endothelial cell adhesion and transmigration (28).

Chronic inflammatory disorder including rheumatoid arthritis, obesity, diabetes, and metabolic syndrome has been considered as a major risk factor of ASCVD, independent of traditional ASCVD risk factors. Driven by systemic inflammation, a remarkable feature of these chronic inflammatory conditions is an enhanced myelopoiesis and a sustained over-activation of the innate immune system, which could in turn contribute to atherosclerosis (84, 85). This is consistent with the induction of trained immunity which is initiated by functional reprogramming of bone marrow progenitor cells and suggests that trained immunity at a systems level may mediate the increased incidence of ASCVD in chronic inflammatory conditions. For example, rheumatoid arthritis patients have been known to be susceptible to atherosclerosis (86). A recent preclinical study reported that systemic inflammation involved in rheumatoid arthritis promotes sustained expansion of HSPCs and myelopoiesis, which is associated with remodeling of cellular cholesterol metabolism (87). These modulations of HSPCs, as conferred by chronic inflammation, eventually lead to enhanced atherosclerosis (87). Interestingly, HSPCs in the context of rheumatoid arthritis is stimulated in a manner very similar to β-glucan-induced training at the level of bone marrow (33). In line with these preclinical data, in patients suffering from rheumatoid arthritis, there is also an enhanced skewing of hematopoiesis toward myeloid lineage as evidenced by a prominent expansion of total circulating monocytes (87). The resulting increased abundance of monocytes are highly enriched in the inflammatory subsets that have been associated with ASCVD, indicating that mature myeloid cells that arise from reprogrammed HSPCs display an enhanced pro-inflammatory state (87). Besides, a cellular cholesterol dysregulation is also present in these rheumatoid arthritis patients that could contribute to atherosclerosis as well (87). Collectively, these findings suggest that chronic inflammatory disorder may result in a prolonged activation of the innate immune system through mechanisms relevant to trained immunity, thus in turn increases the susceptibility to atherosclerosis. During innate immune activation in the context of chronic inflammatory disorder, the associated pro-inflammatory cytokines serve as the endogenous mediator that induces long-term functional reprogramming of bone marrow progenitors (87, 88). One of the key cytokines is IL-1β, which has been established to be of crucial importance for the induction of trained immunity (33, 34). Therapeutic targeting of IL-1β using a monoclonal antibody has yielded success in reducing the incidence of recurrent cardiovascular events in patients experiencing a high risk of ASCVD, which is independent of plasma lipid lowering (6), further indicating that trained immunity may play a causal role in the pathogenesis of ASCVD.

Of note, although trained immunity has been hypothesized to drive atherosclerosis (8, 13–17), currently there is no direct evidence showing that trained immunity is indeed responsible for atherosclerosis development. This is partly due to many pathways involved in innate immune training can exert pro-atherogenic function that is independent of trained immunity, making it difficult to dissect the actual contribution of trained immunity to atherosclerosis. For example, NLRP3 inflammasome is a crucial mediator for WD-induced trained immunity that could potentially promote atherosclerosis (34), but it can also drive atherosclerosis development in a trained-immunity-independent manner (4). Moreover, it is noteworthy that our current mechanistic knowledge about trained immunity are mainly gained from studies using *in vitro* or experimental animal models, clinical translation of these knowledge aimed at targeting trained immunity to treat ASCVD should be cautious. For example, monocytes from patients with symptomatic atherosclerosis, which show a trained immune phenotype, undergo epigenetic rewiring with a reduced histone mark H3K4me3 on pro-inflammatory genes, contrasting an increase of H3K4me3 in the *in vitro* model of trained immunity (31). Besides, while statins targeting cholesterol synthesis pathway has been proved to be effective in preventing trained immunity *in vitro*, it fails to revert the pro-inflammatory state of monocytes from patients with hypercholesterolemia that develop a trained immunity phenotype (83). These findings highlight that trained immunity induced in experimental models may be not equivalent to that induced in human *in vivo* situation in terms of metabolic and epigenetic reprogramming, thus more work is needed to further reveal the molecular mechanisms of trained immunity in human physiological and pathological settings. It is also important to note that the trained cellular states at the molecular and physiological levels are not uniform depending on the initial training stimulus (62). Since patients with atherosclerosis or at a risk of atherosclerosis are exposed to a variety of complex risk factors for ASCVD during their life, there could be a large heterogeneity in terms of trained immunity induced among patients, thus the strategies of targeting trained immunity for the treatment of ASCVD can vary between individuals, and further investigations of trained immunity induced by these various risk factors of ASCVD in patients are warranted.

## Conclusion

**Figure 1:**
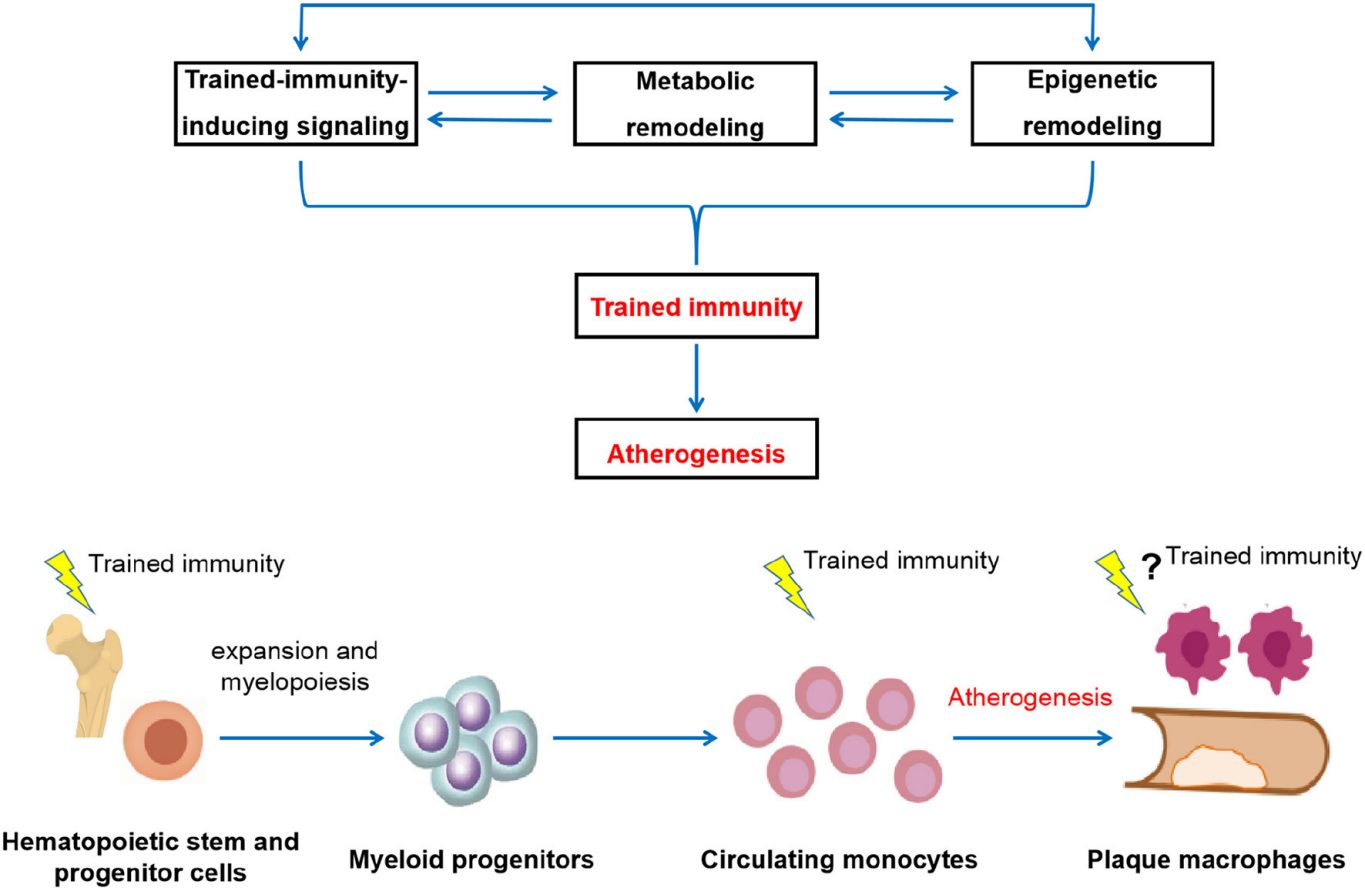
Schematic depictions of the potential pathological relevance of trained immunity in the development of atherosclerosis. The induction of trained immunity is critically mediated by a highly integrated signaling, metabolic, and epigenetic events, including activation of signaling such as IL-1 and GM-CSF pathways, modulation of cellular metabolism such as glycolysis, cholesterol metabolism, fatty acid synthesis, and amino acid metabolism, as well as genome-wide epigenetic rewiring of the histone marks such as H3K4me1, H3K4me3, and H3K27ac. These cellular events are intertwined and highly dependent on each other. Driven by these cellular processes, innate immune cells develop a long-lasting a pro-inflammatory and pro-atherogenic phenotype, thereby contribute to atherosclerosis development (upper panel). In addition to directly act in mature circulating monocytes, training effect can be initiated at the level of bone marrow progenitors, leading to the expansion and modulation of bone marrow progenitor cells and subsequent generation of activated and potentially pathological innate immune cells. Persistent reprogramming of bone marrow progenitors to imprint differentiated innate immune cells for a hyper-responsive state is likely to be a general mechanism for the long-term effect of trained immunity. Through both the cellular level and a systems level, the prolonged over-activation of the innate immune system conferred by trained immunity drives atherosclerosis development (lower panel).

Non-resolving low-grade inflammation mediated by the innate immune system plays a central role in the pathogenesis of atherosclerosis and its related cardiovascular events. Although our understanding of the pathophysiology of atherosclerosis has leapt forward in recent years, the fundamental mechanisms underlying the establishment of non-resolving inflammation characteristic of atherosclerosis remain elusive. Trained immunity, an emerging new concept refers to a prolonged hyperactivation of the innate immune system triggered by various exogenous and endogenous stimuli, has been proposed as a potential contributor for the non-resolving inflammation conducive to atherosclerosis. Here, we review current understanding of the mechanisms that control trained immunity both at the cellular level and a systems level (Tables 1, 2), with a focus on its pro-atherogenic effect and the potential mechanistic relationship between trained immunity and atherosclerosis, and highlight the potential causal role of trained immunity in atherosclerosis development. In summary, trained immunity induction and atherosclerosis development, both of which are dependent on activation of the innate immune system, share common intracellular mechanisms with each other in terms of signaling, metabolic, and epigenetic modulations. Based on current preclinical and clinical data, it is tempting to speculate that trained immunity may be inherently associated with atherosclerosis and could contribute to the development, progression and aggravation of ASCVD (Figure 1). Nonetheless, most current mechanistic insights of trained immunity stem from studies using experimental models with only several model training stimuli which may differ from trained immunity induced in human *in vivo* system, limiting their clinical translation for treating atherosclerosis. Consequently, deeper understanding of the molecular mechanisms of trained immunity and its relevance for atherosclerosis development especially under human pathological conditions will allow the future development of new strategies targeting trained immunity for cardiovascular disease prevention and management.

**Table 1:** A schematic summary of training programs induced in monocytes/macrophages.

**Inducer of training**	**Receptor**	**Trained immunity signaling**	**Metabolic remodeling**	**Epigenetic remodeling**
β-glucan	Dectin-1	AKT-mTOR-HIF1α, cAMP-PKA, IL-1, IL-32	Glycolysis, glutaminolysis, cholesterol/mevalonate synthesis	H3K4me1, H3K4me3, H3K27ac
BCG	NOD2	Akt-mTOR, IL-1 or IFN-γ	Glycolysis, glutaminolysis, cholesterol/mevalonate synthesis	H3K9me3, H3K4me3, H3K27ac
oxLDL	TLR	mTOR-ROS, IL-1	Glycolysis, cholesterol/mevalonate synthesis	H3K4me3
LPS	TLR4	IRAK-1-Tollip, JNK-miR-24-axis	Glucose and cholesterol metabolism	–
Aldosterone	Mineralocorticoid receptor	–	Fatty acid synthesis	H3K4me3

**Table 2:** A schematic summary of training programs induced in bone marrow progenitor cells.

**Inducer of training**	**Trained immunity signaling**	**Metabolic remodeling**	**Transcriptional and epigenetic regulation**
β-glucan	IL-1, GM-CSF/CD131	Glycolysis, cholesterol/mevalonate synthesis	Transcriptional remodeling of myelopoiesis-related genes
BCG	IFN-γ, IL-32	–	Transcriptional remodeling of myelopoiesis-related genes
WD	NLRP3/IL-1	–	Transcriptional and epigenetic remodeling of genes controlling myeloid progenitor cell proliferation and skewing toward the development of activated monocytes

## Author Contributions

CZ and JY designed and drafted the work. XY, YF, and JY reviewed and critically edited the manuscript. All authors read and approved the final manuscript.

### Conflict of Interest

The authors declare that the research was conducted in the absence of any commercial or financial relationships that could be construed as a potential conflict of interest.
